# Quantum chemical calculations of nicotine and caffeine molecule in gas phase and solvent using DFT methods

**DOI:** 10.1016/j.heliyon.2022.e12494

**Published:** 2022-12-20

**Authors:** Ramesh Rijal, Manoj Sah, Hari Prasad Lamichhane, Hari Shankar Mallik

**Affiliations:** a Physics Department, St. Xavier’s College, Kathmandu, Nepal; b Central Department of Physics, Tribhuvan University, Kirtipur, Kathmandu, Nepal

**Keywords:** DFT, HOMO-LUMO, Energy gap, Vibrational spectroscopy, TED assignment

## Abstract

In this study, the molecular structures of nicotine and caffeine molecule have been generated using the 6-311++G(d,p) basis set in the DFT/B3LYP method. The molecules were optimized on the same basis set and their minimum stable energy was calculated. The HOMO-LUMO energies were calculated to establish the kinetic stability and chemical reactivity of the chosen compounds. The variation of energy and its gap were closely studied for both nicotine and caffeine in the presence of solvent water as well. Similarly, vibrational spectroscopy was studied at the most prominent region in both gas phase and solvent water with their respective TED assignments. The shifting of frequency clearly indicates the impact of solvent water and isotopic substitution of carbon atoms.

## 1 Introduction

Caffeine and nicotine are the most common stimulant drugs used globally [1]. These drugs have a significant impact on society and health [2]. People consume these drugs on a daily basis in coffee, tea, dairy products, cold drinks or chocolate [3]. The consumption of these drugs affects the human body physiologically [4].

Caffeine (1,3,7-Trimethyl-3,7-dihydro-1H-purine-2,6-dione) is a natural stimulant [5]. The caffeine content in coffee and tea is approximately 54% and 43%, respectively [6]. Caffeine in high concentration causes faintness, transient cognitive impairment, headaches, and death because of its low boiling point and ease of solubility in water [7]. Most drugs contain caffeine with a low concentration, which stimulates the nervous system, increases alertness, and enhances the high performance of mental activity [8, 9]. Similarly, nicotine (3-(1-methly pyrrolidin-2-yl) pyridine) is a water soluble organic compound with molecular formula C_10_H_14_N_2_ [10]. The addictive nature of nicotine causes paralysis of nervous system [9]. It has impact on cells, the immune system and DNA which might lead to cancer [11].

Many researchers have been working on these compounds for a long time. Still, there has been no comprehensive theoretical investigation of the nicotine and caffeine molecule comparatively in such a larger basis set with such proper simplification and approach in gas phase and water. The main focus of this study is to highlight the molecular structure, vibrational spectroscopy, DOS spectrum and HOMO-LUMO gap accompanying their global reactivity descriptors in both gas phase and solvent water theoretically. Similarly, UV-Vis absorption spectra and contour plot of total density of the chosen molecules have been drawn. Also, the variation of frequencies on vibrational studies while adding solvent and on isotope labeling of carbon has been studied distinctly.

## 2 Methodology

The required chemical calculations were computed in the Gaussian09W program package [12] employing the 6-311++G(d,p) basis set. The calculations were performed using density functional theory (DFT) and the Becke-Lee-Yang-Parr (B3LYP) functional [13, 14]. These methods, functional and program mentioned were used to create the optimized molecular structure and vibrational wavenumbers of the given compounds. The visualization and analysis of the title molecules were done using the Gauss View 5.0 [15]. The geometrical configuration of the title compounds were optimized to account their minimum total energies and were re-optimized for further calculations. The energy gap and global reactivity descriptors were then determined using the same optimized structures for both gaseous and solvent phase. To analyze and plot the DOS spectrum, Gauss Sum program was used [16]. In this study, one of the most widely used and reliable polarizable continuum model (PCM) was used to find the molecular free energies and their corresponding properties in the solvent water [17]. Also, the TD-DFT approach with same basis set was employed to represent the UV-Vis Absorption spectra with its maximum wavelength and oscillator strength. The same basis set and methods were employed to generate the vibrational frequency assignments and various modes of vibration were examined in the gas phase, isotope labeling of carbon and within solvents. The total energy distribution (TED) analysis was evaluated by using localized symmetry in the VEDA4 software [18].

## 3 Result and discussion

### 3.1 Geometry of molecular optimization

**Figure 1:**
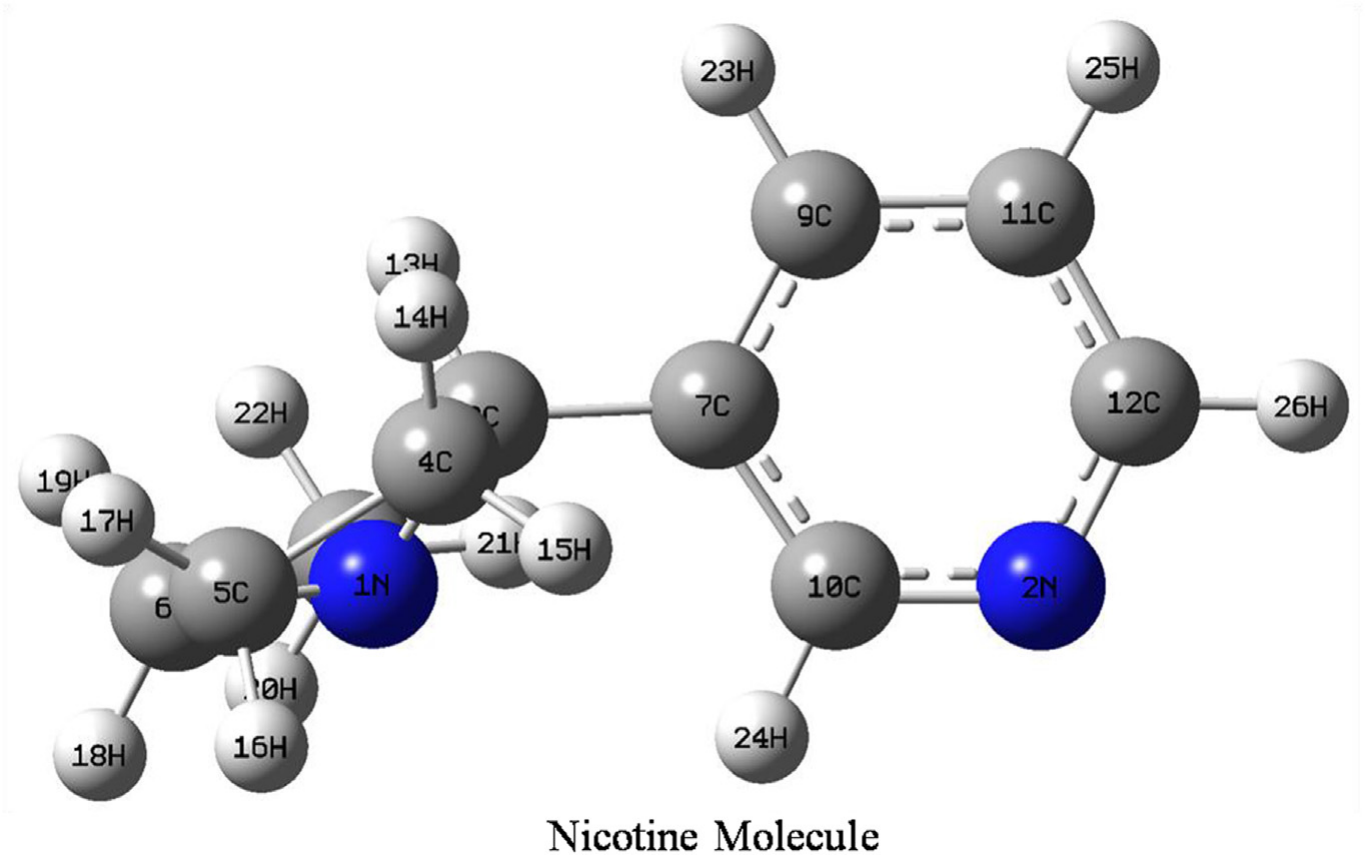
Optimized structure of nicotine molecule with symbol and labeling of atoms.

**Figure 2:**
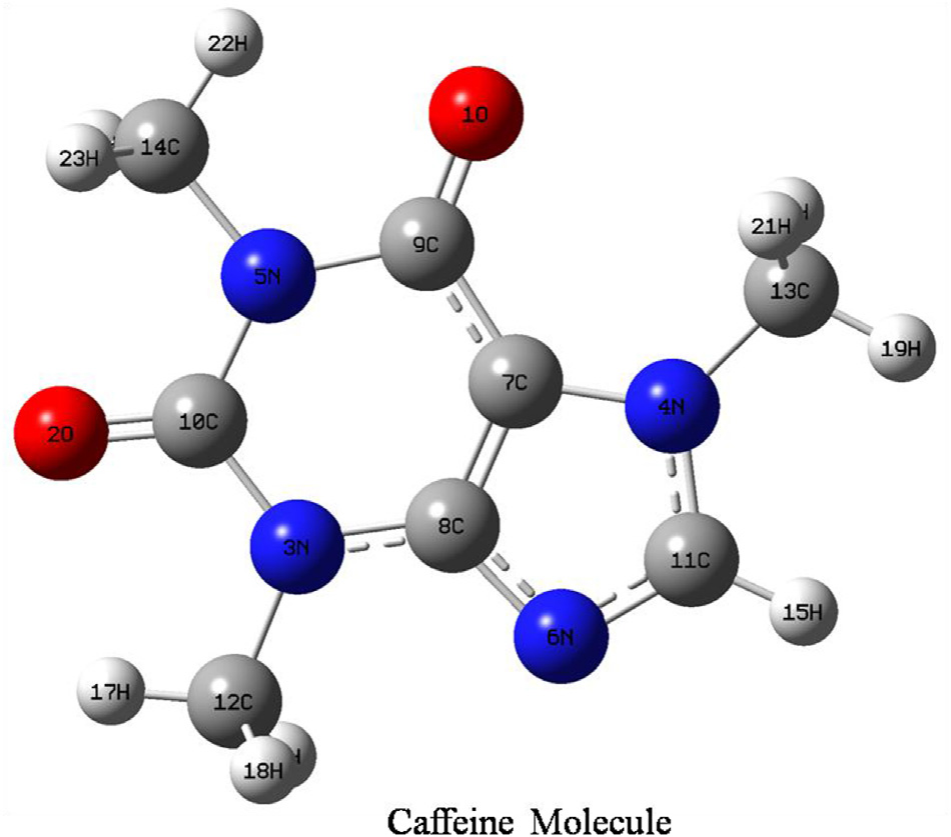
Optimized structure of caffeine molecule with symbol and labeling of atoms.

The minimum total energies of the nicotine and caffeine compounds are calculated by optimizing the respective molecules using the Gaussian 09W program. The optimized geometries of the nicotine and caffeine molecules are presented in Figures 1 and 2, along with their symbols and labeling of atoms.

Table 1 lists all the minimum total energies of nicotine and caffeine molecule with their respective dipole moments both in gas phase and solvent water. The minimum total energy for nicotine molecule in gas phase and solvent water was found to be -13581.43 eV and -13581.6 eV respectively. Similarly, small variation of minimum total energy was observed in caffeine molecule. In the study performed by Mollaamin et al. (2011), values of the dipole moment in the gas phase, solvent water, and methanol reveal that the dipole moment can be enhanced by raising the dielectric constant [19]. It can be clearly observed that the dipole moment was enhanced in solvent water in both molecules. It clearly signifies the increase in polarity of the molecule in the presence of solvent.

**Table 1:** Description of minimum total energy and their dipole for nicotine and caffeine molecule in gaseous phase and water.

State	Minimum Total Energy (eV)	Dipole (Debye)
Nicotine Gas phase	-13581.43	2.97
In solvent Water	-13581.6	4.34
Caffeine Gas phase	-18518.8	3.98
In solvent Water	-18519.21	5.46

### 3.2 HOMO-LUMO analysis

**Figure 3:**
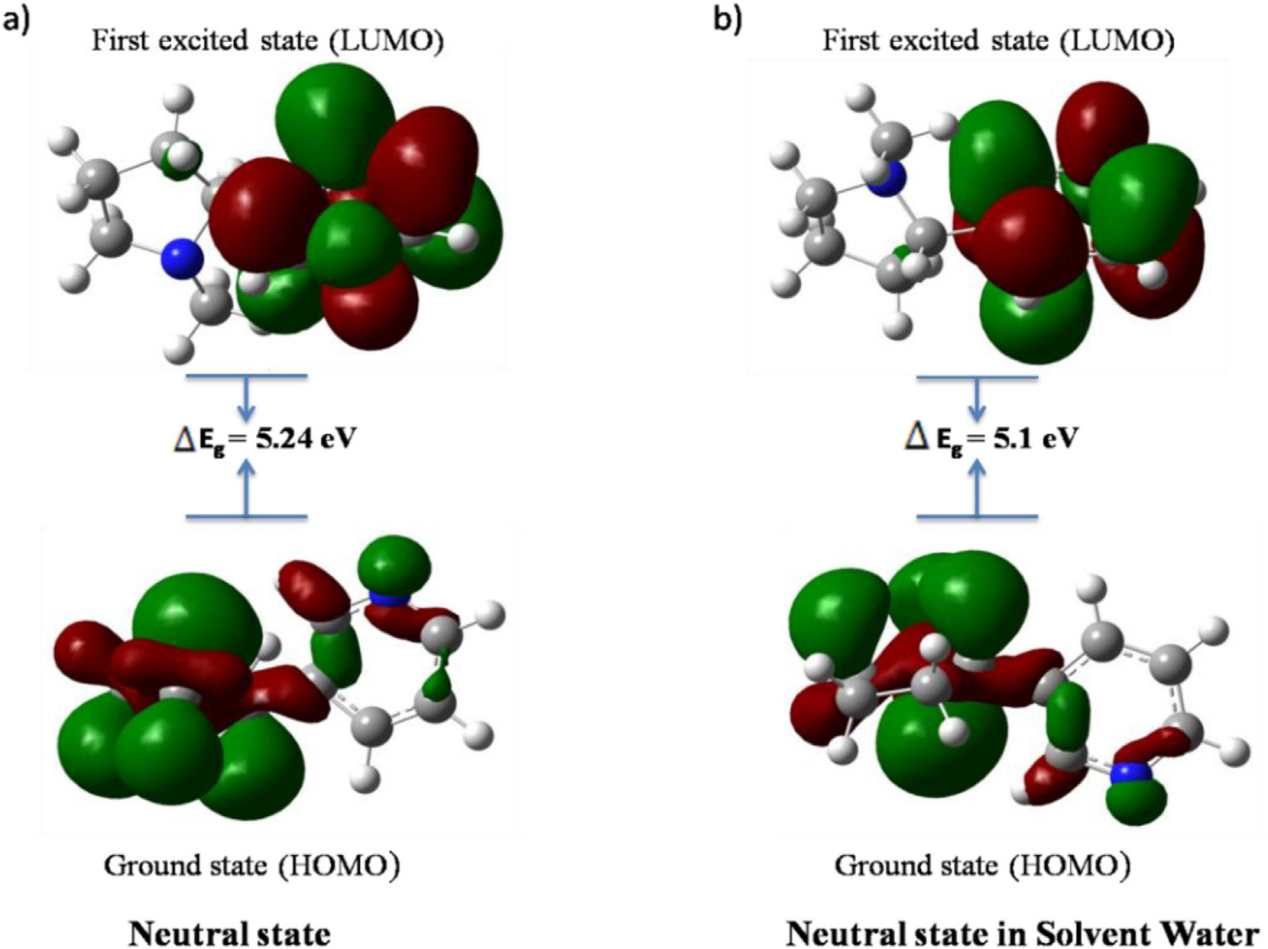
HOMO-LUMO gap of nicotine molecule in a) gas phase and b) solvent water.

**Figure 4:**
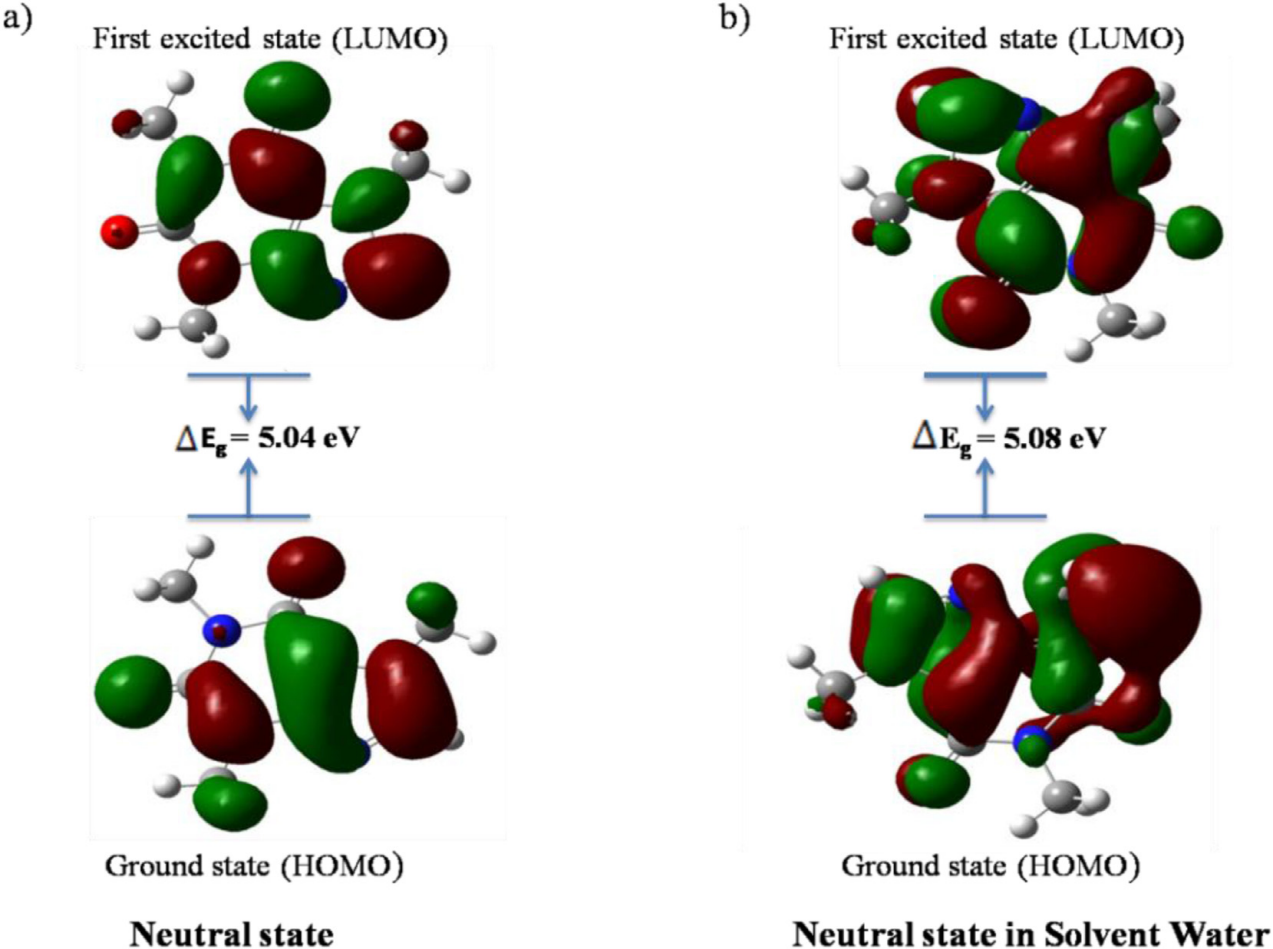
HOMO-LUMO gap of caffeine molecule in a) gas phase and b) solvent water.

The excited state energy of a molecule is represented by a small gap with a manifold called HOMO-LUMO [20]. The energy gap indicates the chemical reactivity of a molecule [21]. HOMO (highest occupied molecular orbitals) and LUMO (lowest unoccupied molecular orbitals) make orbital energy gaps for chemical stability. The energy gap exhibits the charge transfer within the compound [22]. Molecules with the highest electrons in HOMO orbital can easily detach electrons, whereas lowest electron in LUMO orbital easily gains electrons [23]. The frontier molecular orbitals of nicotine and caffeine molecules are calculated in the gas phase and solvent water using the 6-311++G(d,p) basis set, as displayed in Figures 3(a,b) and 4(a,b) respectively. The green and red indicate the positive and negative states, respectively.

Table 2 shows the HOMO and LUMO energies of Nicotine and Caffeine molecules, as well as their energy gaps, in gas phase and solvent water. In the gas phase of neutral state, nicotine molecule has a larger energy gap than in the solvent water. However, in caffeine molecule, the energy gap is larger in the solvent water than in the gas phase. The nicotine molecule has a higher energy gap compared to the caffeine molecule which also coordinates well with the separate study performed by Kurgat et al. 2016 and Salihović et al. 2014 [24, 25]. To conclude, a large energy gap reflects higher kinetic stability and lower chemical reactivity.

**Table 2:** Calculated energy values of HOMO-LUMO (in eV) of nicotine and caffeine molecule.

Parameters	Nicotine Molecule		Caffeine Molecule	
	Gas Phase	In solvent Water	Gas Phase	In solvent Water
HOMO (eV)	-6.2	-6.21	-6.35	-6.41
LUMO (eV)	-0.96	-1.11	-1.31	-1.33
Energy gap (eV)	5.24	5.1	5.04	5.08

### 3.3 Global reactivity descriptors

Using Koopman’s theorem for closed-shell molecules, the parameters such as electron affinity (A), ionization potential (I), chemical hardness (η), chemical softness (S), electronic chemical potential (μ) and global electrophilicity index (ω) [26, 27, 28] of the Nicotine and Caffeine molecule are calculated. The formulae required to calculate them are as follows:

(1) $$\eta =\frac{1}{2}\left(I-A\right)$$

(2) $$S=\frac{1}{\eta }$$

(3) $$\mu =-\frac{1}{2}\left(I+A\right)$$

(4) $$\omega =\frac{\mu^{2}}{2\eta }$$

HOMO-LUMO energy can be used to derive various parameters such as chemical softness, hardness and electrophilicity index. Compounds with a higher HOMO-LUMO gap are more stable and therefore chemically harder than those with a lower HOMO-LUMO gap [29]. Thus, it is certain from Table 3 that nicotine is harder and more stable (less reactive) in gas phase and solvent water, respectively, while caffeine is softer than nicotine and the least stable of all in gas phase (more reactive).

**Table 3:** Global reactivity descriptors of nicotine and caffeine molecule in gas phase and solvent water.

Parameters	Reactivity Descriptor Values (eV)			
	Nicotine Molecule		Caffeine Molecule	
	Gas Phase	In Solvent Water	Gas Phase	In solvent Water
Electron Affinity (A)	0.96	1.11	1.31	1.33
Ionization Energy (I)	6.2	6.21	6.35	6.41
Chemical Hardness (η)	2.62	2.55	2.52	2.54
Chemical Softness (S) (eV)^−1^	0.38	0.39	0.4	0.39
Electronic Chemical Potential (μ)	-3.58	-3.66	-3.83	-3.87
Global electrophilicity index (ω)	2.44	2.63	2.91	2.94

According to the global electrophilicity index, organic compounds can be categorized as strong electrophiles with ω > 1.5 eV, moderate electrophiles with ω between 0.8 eV and 1.5 eV, and marginal electrophiles with ω < 0.8 eV [30]. In our study, both molecules have strong electrophiles in both gaseous phase and solvent water, with the caffeine molecule having the highest value of 2.94 eV in the presence of solvent water. Besides, the electron affinity and ionization potential is higher when the solvent water is added.

### 3.4 Density of states and total density

**Figure 5:**
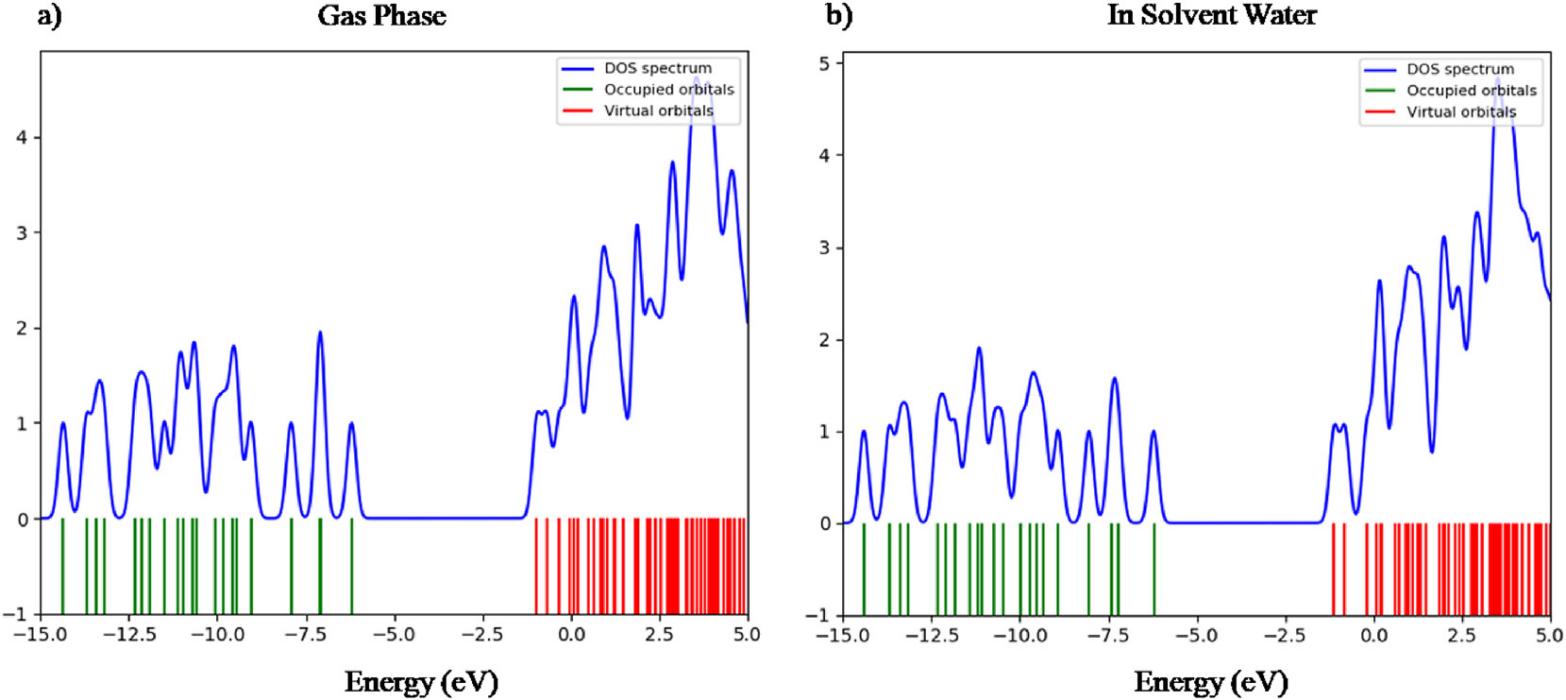
Density of states of nicotine molecule in a) gas phase & b) solvent water.

**Figure 6:**
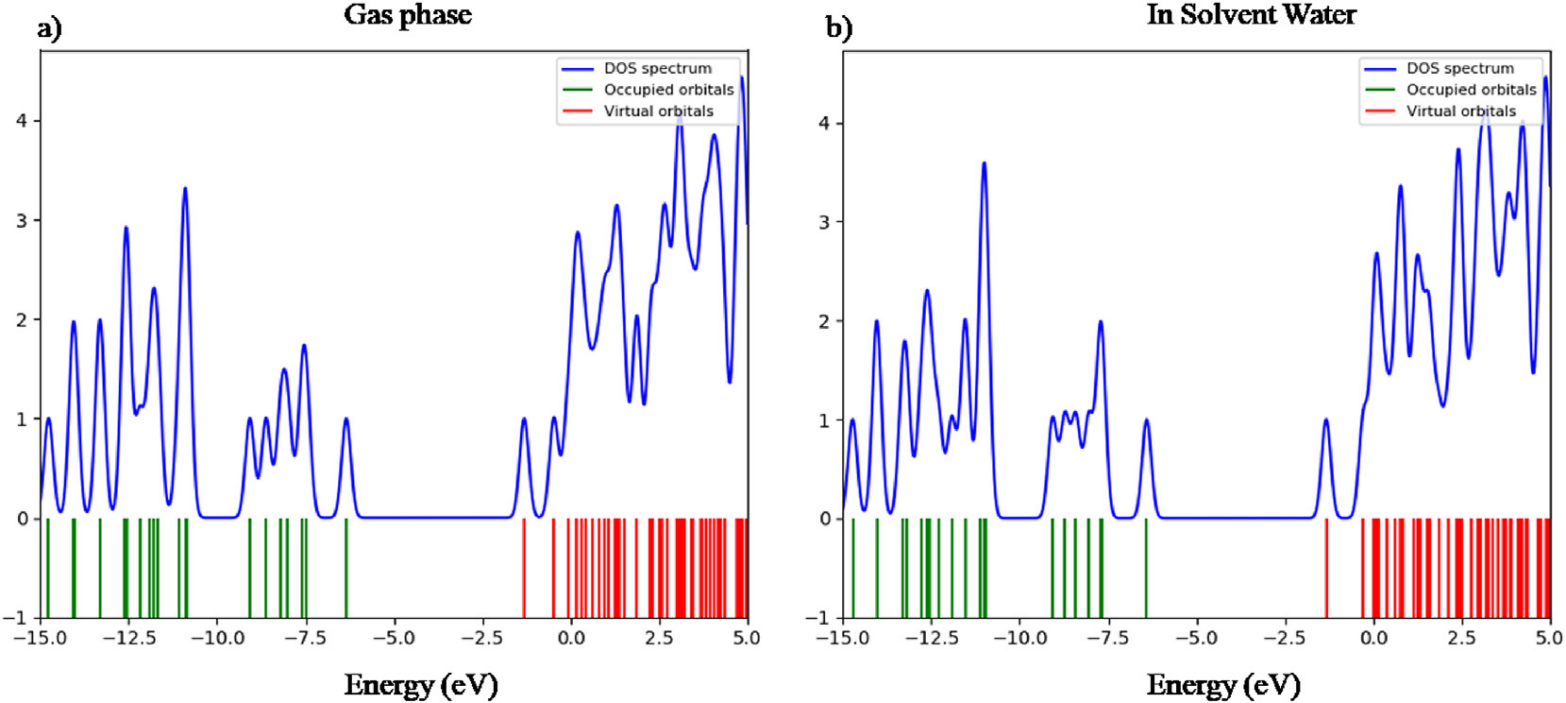
Density of states of caffeine molecule in a) gas phase & b) solvent water.

The stimulation of electron from valence band to conduction band is performed by using DOS (Density of states) spectrum [31]. The DOS spectrum was generated with a full width at half maximum (FWHM) of 0.3 eV by operating the Gauss Sum 3.0 program [16]. It clarifies the significance of electrons to the conduction and valence band. The DOS spectra in Figures 5 and 6 clearly explain the availability of multiple states at different energy levels.

The density of states of nicotine and caffeine molecule is presented in Figures 5(a,b) and 6(a,b) respectively for gas phase and solvent water. A positive value on the DOS indicates a bonding interaction, a negative value indicates an anti-bonding interaction, and a zero value signifies no bonding interaction [32]. A high-intensity DOS at distinct energy levels suggests the availability of multiple states for occupation. The HOMO-LUMO gap and the energy gap depicted in the spectrum of DOS are equivalent and well coordinated with each other.

**Figure 7:**
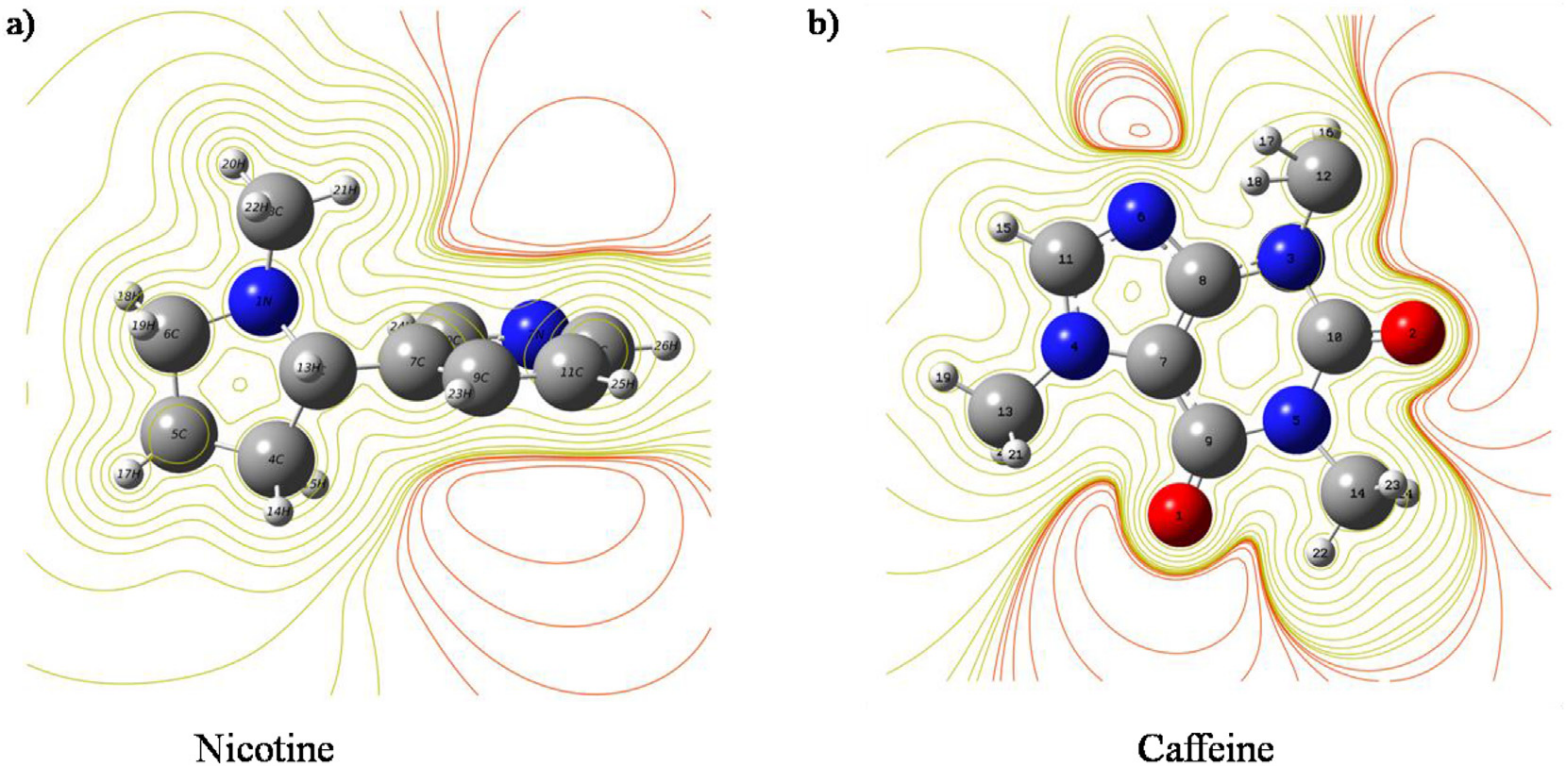
Contour plot of molecular electrostatic potential surface of a) Nicotine and b) Caffeine molecule.

The lines in the contour plot vividly demonstrate the path of electron density in the chosen molecules presented in Figure 7a and b respectively. The red color area signifies the negative zone where the oxygen atom is mostly surrounded. Similarly, other colored regions with the maximum dominating area represent the positive zone of the title molecules. The carbon atom in the methyl group is naturally negatively charged to a greater extent than other similar atoms since it is sharing electrons with three hydrogen atoms.

### 3.5 Molecular electrostatic potential (MEP) analysis

**Figure 8:**
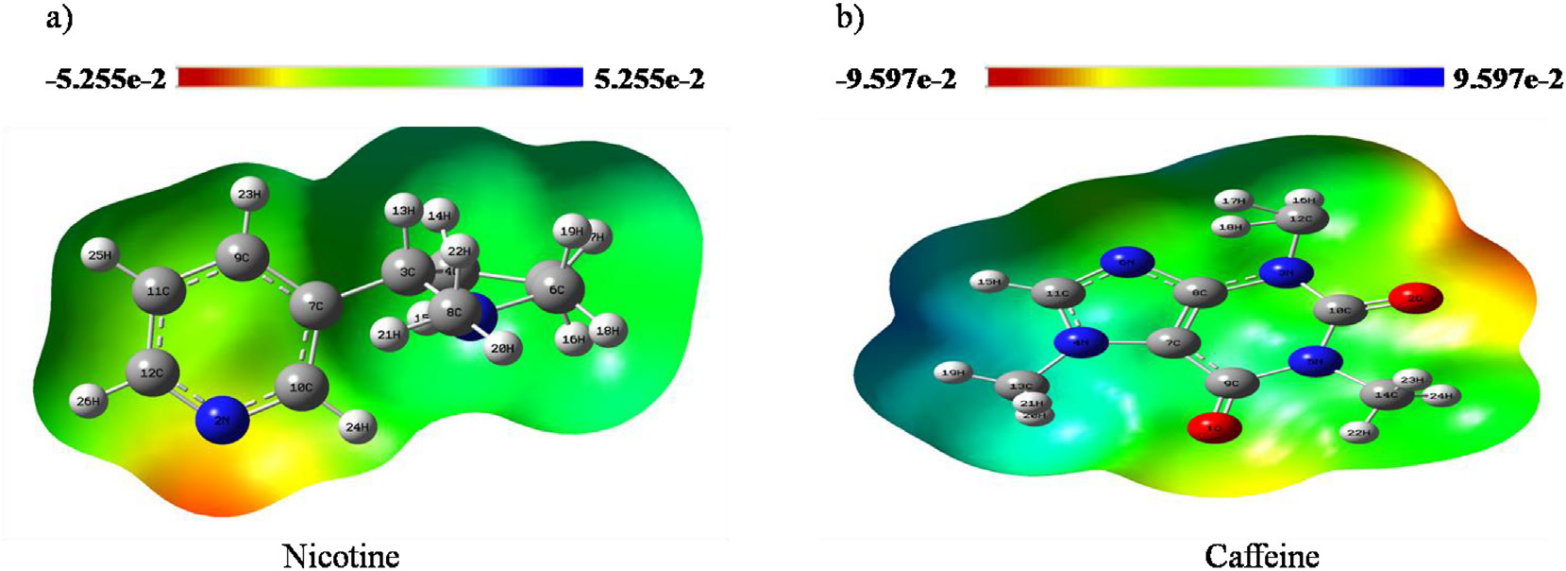
Molecular Electrostatic Potential map of a) Nicotine and b) Caffeine.

MEP plays a significant role in understanding the electrophilic and nucleophilic sites of a given compound [33]. The maximum positive region represented by blue color is the favored site for nucleophilic attack, and the negative region with red color is the favored site for electrophilic attack [34]. The potential represented in terms of color grading follows the order: red < orange < yellow < green < blue as represented in Figure 8. The MEP maps of the nicotine and caffeine molecules have been presented in Figure 8a and b, respectively. The negative and positive potential ranges from -5.255e-2 a.u. to 5.255e-2 a.u. and -9.597e-2 a.u. to 9.597e-2 a.u. for Nicotine and Caffeine, respectively. In our study, most of the regions are neutral in nature for both nicotine and caffeine. The nitrogen atom of nicotine and two oxygen atoms of caffeine molecule are confined to negative region. Mostly, electrophilic attack is favored in both the molecules.

### 3.6 Mulliken atomic charges

**Figure 9:**
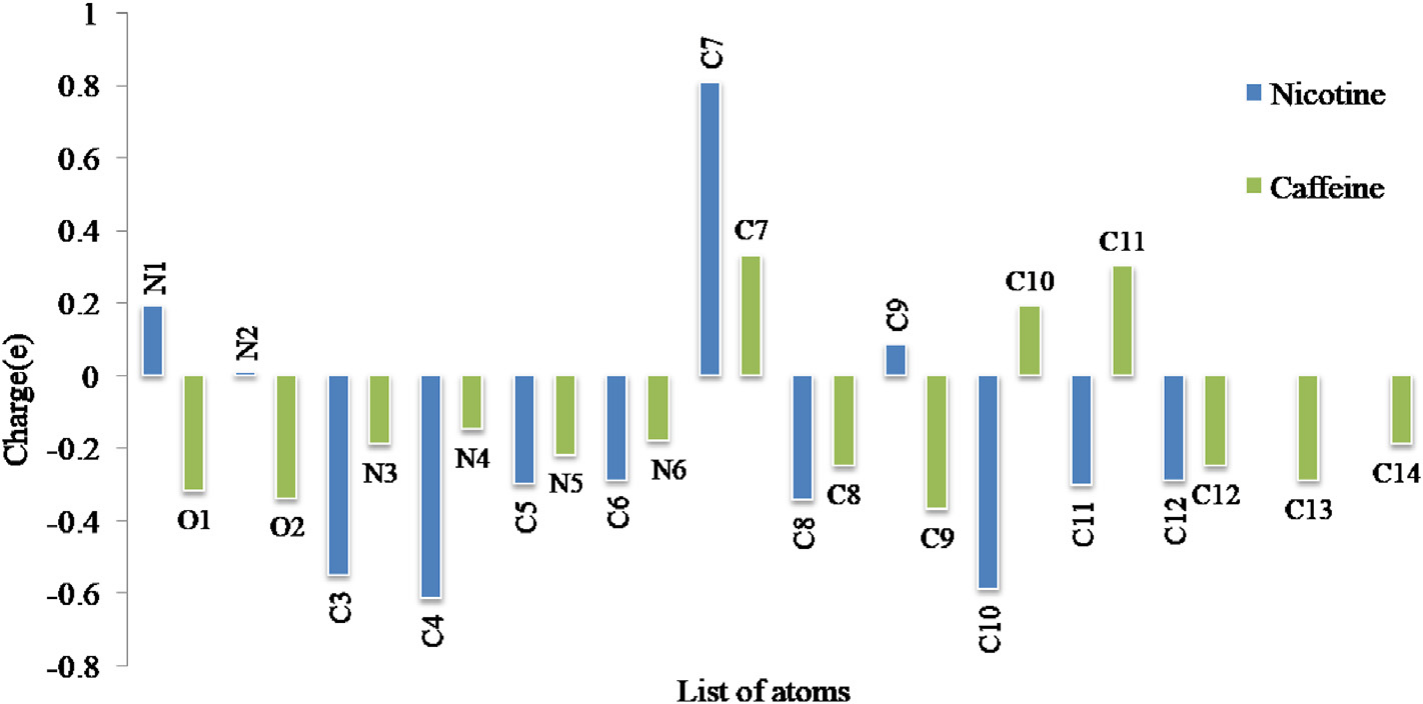
Mulliken charge distribution of nicotine and caffeine molecule.

Mulliken atomic charges are vital in quantum chemical calculations as they have a significant impact on molecular characteristics, electronic structure, polarizability, etc [35]. The Mulliken atomic charges are presented in Figure 9. The C7 atom has the highest positive charges both in nicotine and caffeine. The negative charges are found in the oxygen atoms of both molecules. The nitrogen and carbon atoms have both positive and negative charges. Since every hydrogen atoms have a minute positive charge, they are not included in the figure.

### 3.7 Vibrational analysis

The nicotine and caffeine molecules are composed of 26 and 24 atoms having 72 and 66 modes of vibration respectively. No imaginary modes of vibration have been observed during the frequency calculation, and the wavenumbers were generated only after complete optimization at the true minimum potential energy surface. The modes of vibration occurring between the intensive regions of 1650 and 1100 cm^−1^ were investigated with their total energy distribution (TED) assignment for gas phase, in the presence of solvent, and isotope labeling of carbon, respectively.

**Figure 10:**
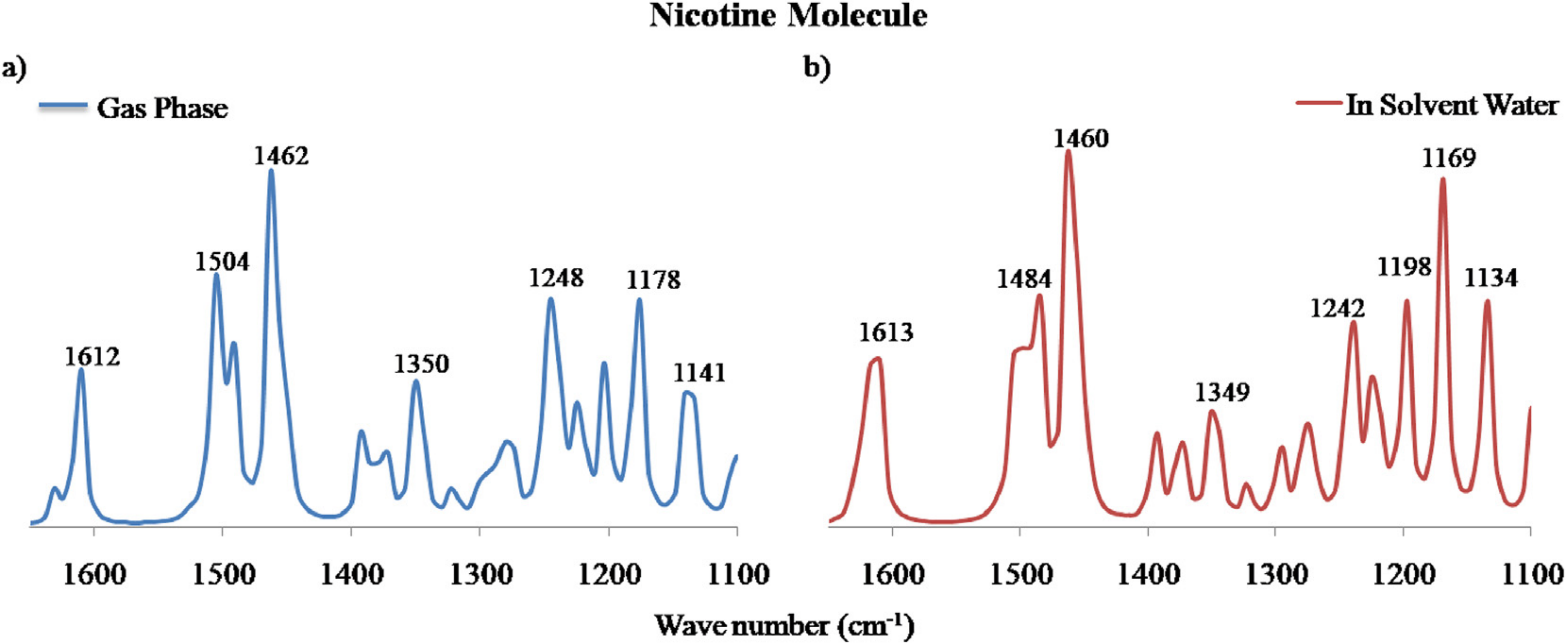
Calculated FT-IR spectra of nicotine molecule in the range of frequency 1650–1100 cm^−1^ in a) gas phase and b) solvent water.

Normally, C–C stretching modes of vibration are expected to occur in the region of 1625-1450 cm^−1^ [36]. In our investigation, FT-IR band was identified at 1612 and 1613 cm^−1^ in gas phase (Figure 10a) and solvent water (Figure 10b) of nicotine molecule with respective TED assignment of 29% and 26%. Similarly, in caffeine, it was found at 1620 cm^−1^ and 1624 cm^−1^ respectively. During the isotope labeling of carbon, the values were lowered to 1560 and 1574 cm^−1^ in nicotine and caffeine which can be observed in Figure 12a and b respectively.

**Figure 11:**
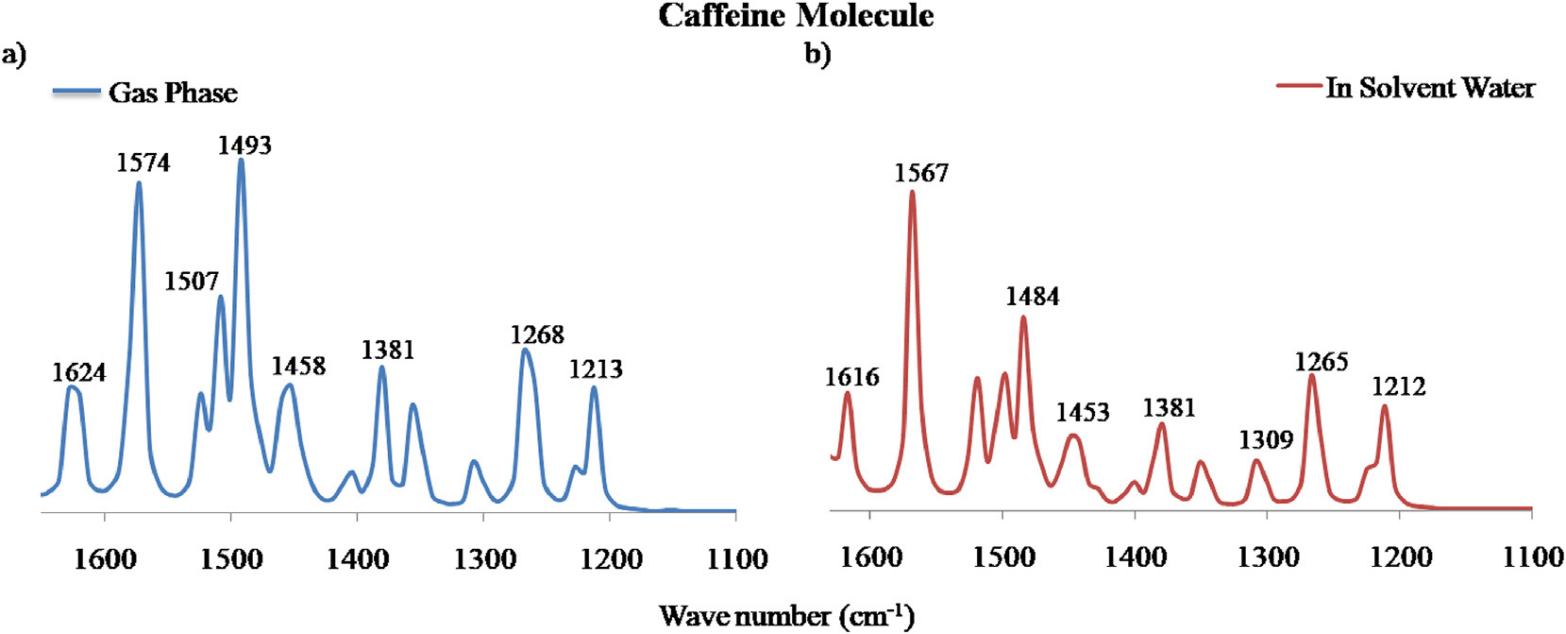
Calculated FT-IR spectra of caffeine molecule in the range of frequency 1650-1100 cm^−1^ in a) gas phase and b) solvent water.

Usually, C–N stretching vibrations occur at around 1600-1300 cm^−1^ and might vary due to possible mixing of several bands [37]. In case of nicotine molecule, the C–N vibration was evaluated at 1630 cm^−1^ and 1626 cm^−1^ with TED assignment of 24% and 25%. Likewise, in caffeine, it was observed for gas phase (Figure 11a) and solvent water (Figure 11b) at 1353 cm^−1^ and 1359 cm^−1^ respectively. On isotope labeling, the down shifting was around 50 cm^−1^ for both the molecules. The theoretical observation in our study is in consistent with the experimental results performed by Visak et al. (2011) on nicotine and Ucun et al. (2007) on caffeine molecule [38, 39].

**Figure 12:**
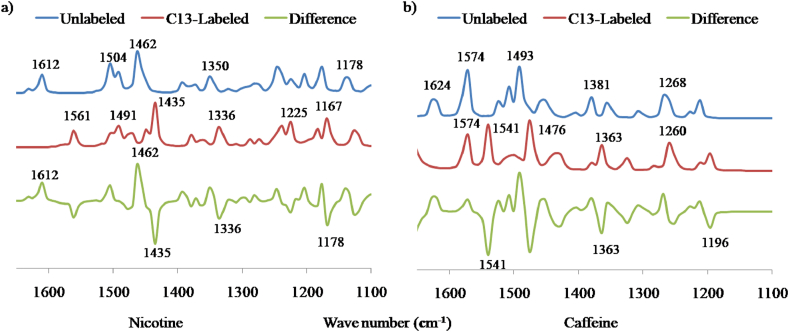
Calculated FT-IR spectra with unlabeled, C13-labeled and their difference in the range of frequency 1650–1100 cm^−1^ for a) Nicotine and b) Caffeine Molecule.

Similarly, various bending and torsion vibrations were observed and their corresponding frequencies and TED contributions have been highlighted in Tables 4, 5, and 6. As mentioned, bending or deformation vibration has been assigned to various angles between three atoms and torsion vibration has been observed at various dihedral planes or angles. It is obvious that on isotopic substitution of a larger mass, the frequency shifts downward [40]. The down shifting of frequency in our study ranges from 5–60 cm^−1^ which is clearly noticeable in Figure 12.

**Table 4:** Vibrational assignment of calculated frequencies along with their TED contribution in gas phase and solvent water for nicotine molecule.

Modes of Vibration	IR Frequency in Neutral state with TED% of Nicotine Molecule			
	Gas Phase	TED%	In solvent Water	TED%
v(N2–C10)	1630	24	1626	25
v(C12–C11)	1612	29	1613	26
v(N1–C8)	1178	29	1169	35
δ(H14–C4–C5)	1248	22	1242	20
δ(H17–C5–H16)	1490	38	1498	33
δ(H21–C8–H20)	1493	39	1484	28
τ(H14–C4–C5–C6)	1298	18	1297	16
τ(H22–C8–N1–C3)	1504	11	1492	13

v, δ and τ denote stretching, bending (deformation) and torsion modes of vibration.

**Table 5:** Vibrational assignment of calculated frequencies along with their TED contribution in gas phase and solvent water for caffeine molecule.

Modes of Vibration	IR Frequency in Neutral state with TED% of Caffeine Molecule			
	Gas phase	TED%	In solvent Water	TED%
v(N6–C11)	1353	20	1349	23
v(C7–C8)	1624	50	1620	50
δ(H15–C11–N6)	1213	31	1196	27
δ(H21–C13–H20)	1524	49	1520	41
δ(C7–C8–N6)	1574	26	1567	25
τ(H17–C12–N3–C10)	1154	35	1149	39
τ(H24–C14–N5–C10)	1268	13	1260	12

**Table 6:** Vibrational assignment of calculated frequencies for unlabeled and isotope labeling of carbon in nicotine and caffeine molecule.

Modes of Vibration	IR Frequency in Nicotine		Modes of Vibration	IR Frequency in Caffeine	
	Unlabeled	C13 Labeled		Unlabeled	C13 Labeled
v(C12–C11)	1612	1560	v(C7–C8)	1624	1574
τ(H22–C8–N1–C3)	1504	1500	δ(C7–C8–N6)	1574	1541
δ(H26–C12–N2)	1462	1449	δ(H16–C12–H18)	1507	1495
δ(H24–C10–N2)	1350	1336	δ(H22–C14–H24)	1493	1476
δ(H14–C4–C5)	1248	1237	v(N4–C11)	1381	1363
δ(H16–C5–C6)	1203	1185	τ(H24–C14–N5–C10)	1268	1260
v(N1–C8)	1178	1167	δ(H15–C11–N6)	1213	1196

### 3.8 UV-Vis Absorption Spectra

**Figure 13:**
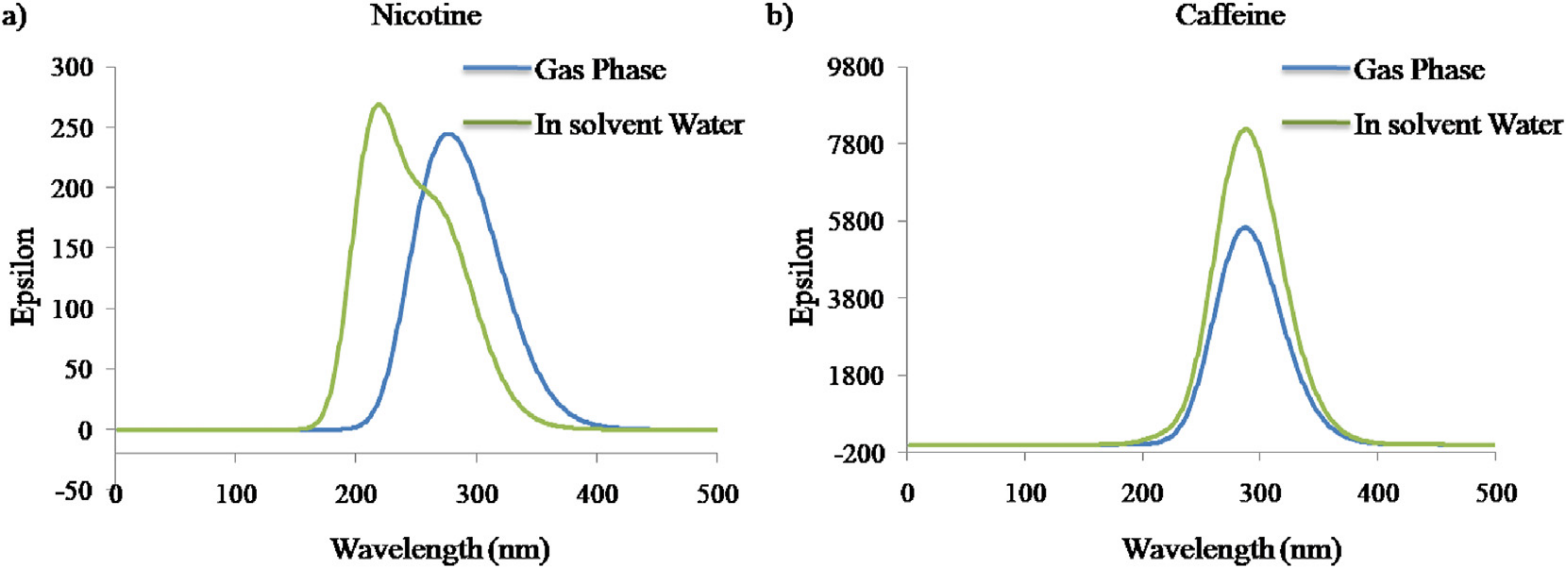
UV-Vis Absorption Spectra for a) nicotine molecule and b) caffeine molecule in gas phase and solvent water.

The ultraviolet-visible (UV-Vis) spectra of nicotine and caffeine obtained using the TD-DFT method in gas phase and solvent water at 6-311++G(d,p) basis set is depicted in Figure 13. The maximum wavelength, oscillator strength, and excited state energies are presented in Table 7. The C-PCM model was proposed to analyze the impact of solvent. The presence of solvent in caffeine has a minor impact, but noticeable change can be observed in Figure 13a for nicotine. The maximum peak absorbance for the nicotine molecule in the gas phase slightly differs from the experimental results calculated from the mainstream of smoking cigarettes [41]. Similarly, the maximum peak absorbance for the caffeine molecule (Figure 13b) observed in the solvent water is quite similar to the experimental results reported by Belay et al. (2008) [42].

**Table 7:** UV-Vis excitation energy (eV) and oscillator strength (f) with max wavelength (nm).

State	Max. Wavelength (nm)	Experimental Value (nm) [41, 42]	Excitation Energy (eV)	Oscillator strength (f)
Nicotine Gas phase	255.58	220	4.85	0.0041
In solvent Water	248.05	--	4.99	0.0061
Caffeine Gas phase	270.23	--	4.59	0.1388
In solvent Water	270.34	272	4.58	0.2020

## 4 Conclusions

This research work involves theoretical investigation of nicotine and caffeine molecule in terms of their stability, reactivity, and interaction in gas phase and solvent water. The minimum stable energy was found in the gas phase and the dipole was found higher in presence of solvent water for both nicotine and caffeine. The nicotine molecule in gas phase and the caffeine molecule in solvent phase show a higher energy gap, making them more kinetically stable. Caffeine compounds are found to be softer and more electrophilic than nicotine. The spectra of DOS well coordinate with the energy gap depicted in the HOMO-LUMO analysis. The FT-IR spectrum of the chosen molecules was assigned in terms of the TED contribution. The theoretical results obtained in our study are in good agreement with the experimental data from the literature review. Hence, we anticipate that the quantum chemical calculation performed on a larger basis set in our study will be very useful for understanding the overall activity and dynamics of the molecule. These computational findings will also help to design further experiments and predict the outcomes.

## Declarations

### Author contribution statement

Ramesh Rijal: Conceived and designed the experiments; Wrote the paper.

Manoj Sah: Performed the experiments.

Hari Prasad Lamichhane: Analyzed and interpreted the data.

Hari Shankar Mallik: Contributed reagents, materials, analysis tools or data.

### Funding statement

This research did not receive any specific grant from funding agencies in the public, commercial, or not-for-profit sectors.

### Data availability statement

Data will be made available on request.

### Declaration of interests statement

The authors declare no competing interests.

### Additional information

No additional information is available for this paper.
